# Summary of the various treatments for osteonecrosis of the femoral head by mechanism: A review

**DOI:** 10.3892/etm.2014.1811

**Published:** 2014-06-26

**Authors:** CHENG WANG, JIANG PENG, SHIBI LU

**Affiliations:** Institute of Orthopedics, Chinese PLA General Hospital, Beijing 100853, P.R. China

**Keywords:** osteonecrosis, osteoblast, osteoclast, bisphosphonates

## Abstract

Osteonecrosis of the femoral head (ONFH), also known as femoral head avascular necrosis, is a pathological state with a number of possible etiologies including steroid administration, alcohol abuse, traumatic events, vascular injury and idiopathic origins. ONFH causes a reduction in the vascular supply to the subchondral bone of the femoral head, which results in osteocyte death and the collapse of the articular surface. Treatments for ONFH include non-weight-bearing therapy, physical support, the promotion of osteoclast apoptosis, and the reduction of osteoblast and osteocyte apoptosis. The aim of the present review was to summarize the treatments for ONFH by mechanism from a new perspective and to describe the condition with an emphasis on treatment options.

## 1. Introduction

Osteonecrosis of the femoral head (ONFH) results in the collapse of the femoral head and the rapid destruction of the hip joint. ONFH has a significant impact on adults aged between 35 and 55 years. This pathological state has several possible etiologies that cause a reduction in the vascular supply to the subchondral bone of the femoral head, resulting in microcirculation disturbance and the subsequent collapse of the femoral head (1–3). In general, the type of treatment carried out varies according to the disease etiology. Treatments include non-weight-bearing (NWB) therapy, physical support and the alteration of cytoactivity. Extracorporeal shock wave therapy (ESWT) is used to reduce osteoblast and osteocyte apoptosis. Other therapies used include hyperbaric oxygen (HBO) therapy and core decompression. Certain reports (4–6) suggest that bisphosphonates increase osteoclast apoptosis and prevent the collapse of the femoral head. Various femoral head-preserving procedures have been carried out in an attempt to prevent the requirement for a complete hip replacement. The present review focuses on treatments for ONFH (7,8).

## 2. NWB therapy

The initial treatment for early stage ONFH is NWB therapy (9), including a complete rest from weight bearing or changing the weight-bearing status. This type of therapy is able to prevent damage to vessels that supply the femoral head, which is advantageous for ONFH with articular collapse (10). However, a previous review identified that limiting the patient to a wheelchair or walking frame to reduce weight bearing does not achieve satisfactory clinical benefits (11). It was observed that in nonoperatively managed patients with ONFH, only 20% achieved recovery and 80% required surgery, including total hip replacement or other treatments (11).

Transtrochanteric rotational osteotomy was developed in 1972 (12,13) to prevent the progressive collapse of the articular surface and osteoarthritic changes in patients with ONFH. The process is technically demanding and preserves the shape of the femoral head by altering its weight-bearing status (14). However, a number of studies (15–18) have indicated variable rates of success for the surgery. A study by Hiranuma *et al* (16) evaluated hip instability following transtrochanteric rotational osteotomy. Instability was defined as >1 mm translation of the femoral head appearing on transverse computed tomography scans obtained at 0 and 45° flexion of the hip joint; 11 of the 27 hips studied (~40%) revealed instability following surgery.

Although NWB therapy is widely used to treat the early stages of ONFH, certain studies (19–21) have concluded that it is not able to cure the disease or prevent femoral head collapse if it is the only treatment. NWB therapy did not prevent ONFH in rats in a study by Okazaki *et al* (21), which also clarified the role of weight bearing in the development of ONFH. In the study, an ONFH model was established in non-weight-bearing and weight-bearing groups of rats following lipopolysaccharide and methylprednisolone stimulation. Three weeks after the final methylprednisolone injection, the two groups did not differ in the progression of ONFH. Thus, the authors concluded that weight bearing may not contribute to the development of non-traumatic ONFH in rats.

Transtrochanteric rotational osteotomy is an ideal treatment for joint preservation. However, it has not gained widespread acceptance as a treatment for ONFH due to the inconsistency of the obtained results.

## 3. Physical support

### Bone grafting

The ideal surgical procedure for ONFH would be to remove the necrotic bone from the femoral head and replace it with viable and structurally sound bone, thereby restoring vitality to the femoral head and preventing the collapse of the articular surface. Bone grafting is an appealing treatment option as it combines the benefit of decompressing the femoral head with the introduction of osteoconductive and/or osteoinductive material into the devitalized head. Furthermore, such grafting preserves the natural hip geometry and articular cartilage, unlike the osteotomy and arthroplasty methods.

#### Nonvascularized and vascularized bone grafting

Nonvascularized bone grafting is an attractive alternative treatment used when there is precollapse or minimal postcollapse of ONFH with relatively preserved articular cartilage (22–27). Rosenwasser *et al* (28) reported that thorough debridement and cancellous bone-grafting in patients with ONFH was an efficacious treatment, with 87% of the patients studied remaining essentially free of symptoms and with minimal progression of osteoarthritis following the procedure.

Vascularized bone is grafted onto the necrotic femoral head to directly target its devascularized status. Such a procedure, in addition to replacing necrotic bone with healthy bone, establishes a new source of circulating blood, thus introducing osteoinductive cells and promoting the restoration of a healthy subchondral plate (29–31). Tetik *et al* (23) carried out a clinical study comparing the results of vascularized and nonvascularized fibular grafting and revealed more improved clinical and radiographic results with vascularized compared with nonvascularized grafting.

Free vascularized fibular grafting has been successfully used as a joint-preserving procedure treatment for ONFH, and a number of studies have demonstrated satisfactory mid- and long-term outcomes (32–36). The procedure may be effective for avoiding or preventing the requirement for complete hip arthroplasty in young patients with early to intermediate stages of ONFH. Kawate *et al* (37) identified that minor osteonecrosis (<300° of the femoral head) without preoperative collapse (Steinberg classification stages I and II) was the primary indicator for free vascularized fibular grafting treatment. Osteonecrosis induced by steroid use was a relative counter indicator. Major osteonecrosis (>300° of the femoral head), with a severe preoperative collapse (>3 mm), was a major counter indicator. Studies concerning grafting as a treatment for ONFH in patients with lymphoma are rare. The surgical benefits and safety remain to be systematically assessed. Yin *et al* (38) retrospectively reviewed seven patients (14 hips) with lymphoma (two cases of Hodgkin’s disease and five cases of non-Hodgkin’s lymphoma) who underwent free vascularized fibular grafting for ONFH. All patients exhibited a positive recovery without any severe life-threatening complications. The mean Harris hip score improved from 69 to 88, while the mean pain score on a visual analog scale decreased from 54 to 18. Radiography revealed either improved or unchanged results in the three hips with initial Steinberg classification stage II osteonecrosis and in nine of the 11 hips with stage III or IV osteonecrosis. None of the hips demonstrated a failure of the treatment or required complete hip arthroplasty. Despite the technically demanding nature of the procedure, the use of free vascularized fibular grafting improves the quality of life of patients with ONFH, with functional improvement and the alleviation of pain. The procedure involves decompressing the femoral head, excising the necrotic bone and adding a cancellous bone graft with osteoinductive and osteoconductive properties, which augments revascularization and neo-osteogenesis of the femoral head (37,39).

### Tantalum implants

Preservation of an osteonecrotic femoral head relies on preventing the collapse of the structurally compromised necrotic bone. Tantalum rod implantation has been proposed as a treatment in the early stages of ONFH. It has demonstrated bone ingrowth and rapid fixation in functional and non-functional animal models and in human explant case reports (40–43). The procedure preserves the structural integrity of the femoral head and is most effective in hips at the pre-collapse stage.

The use of tantalum rods for ONFH was first proposed in 1997 by Schnieders *et al* (44). The authors used a magnetic resonance-based three-dimensional finite element model of ONFH to study the mechanical effects of inserting a porous tantalum rod into a necrotic femoral head. The porous tantalum rod was a reasonable mechanical substitute for a fibular graft, since it effectively reduced the peak ratio of stress to strain. Liu *et al* (45) demonstrated that a porous tantalum implant effectively supports the subchondral bone of the femoral head and that the strength of the implant is >9-fold than that of the loading stress exerted on the implant. Varitimidis *et al* (43) studied 27 patients who underwent tantalum rod implantation for nontraumatic ONFH. The implant was a porous tantalum rod (10 mm). One patient (one hip) succumbed 15 months following surgery for unrelated reasons. In total, 13 of the 26 hips remained at the same radiographic stage, while 13 revealed deterioration. The authors concluded that porous tantalum rods were simple to use via a minimally invasive and reproducible method, and may provide functional recovery for patients at pre- and post-collapse stages of hip osteonecrosis.

These results confirm the curative effects of a porous tantalum rod prosthesis on ONFH, particularly at Steinberg classification stages I and II and at stage IIIA with minimal collapse. The procedure resolves pain as well as preventing and curing the collapse of the femoral head due to necrosis.

## 4. Increased osteoclast apoptosis and reduced osteoblast and osteocyte apoptosis

A number of studies have proposed a balanced system of manipulating osteoclasts and osteoblasts in order to prevent collapse of the femoral head in patients with ONFH (46,47). Changes in the prevalence of osteoblast apoptosis may have a significant impact on the number of osteoblasts present at bone formation sites and on their function during therapies including ESWT, HBO treatment and core decompression. Furthermore, an increase in osteoclast apoptosis and inhibited action of mature osteoclasts on bone reduces bone diminution and reinforces bone remodeling (48).

### Osteoblast promotion

#### ESWT

ESWT began with the incidental observation of the osteoblastic response pattern during animal studies in the mid-1980s, which generated interest in the potential use of ESWT for musculoskeletal disorders (49). Shockwaves are high-energy acoustic waves generated by electrohydraulic or electromagnetic principles. ESWT is effective for treating non-union long bone fractures and tendinopathy of the shoulder, elbow, knee and heel (50) It has been trialed as a treatment for early ONFH and a number of studies (51) have reported the positive effects of the therapy for ONFH (52,53).

Wang *et al* (52) demonstrated that ESWT is a more effective treatment than core decompression and nonvascularized fibular grafting for patients with early ONFH. In ESWT treatment, the affected hip is positioned in adduction and with internal rotation by securing the limb to a table. For lesions at stages II or III, the junctional zone between the avascular and vascular bone of the femoral head is delineated under C-arm control. Four focal points are selected, ~1.0 cm apart, and marked with a marker. Each of the four points is treated with 1,500 shock wave impulses at 28 kV (equivalent to 0.6 mJ/mm^2^ energy flux density), for a total of 6,000 impulses. Following treatment, patients are instructed to walk on crutches with partial weight bearing on the affected limb for 4–6 weeks. Yin *et al* (50) reported that ESWT significantly enhanced the angiogenic and osteogenic effects of bone-marrow stem cells via the nitric oxide pathway in patients with ONFH. Thus, ESWT is a novel non-invasive alternative to surgery (which therefore avoids the risks associated with surgery) and is increasingly clinically applied.

#### HBO therapy

HBO has numerous treatment implications as it has a number of physiological and pharmacological modes of action. Various studies have presented histological evidence that the earliest stage of ONFH is preceded by bone-marrow edema, as observed on magnetic resonance imaging (MRI) scans (54,55). Venous drainage is restored by reducing intraosseous pressure, thereby improving the microcirculation. HBO restores tissue oxygenation, reduces edema and induces angioneogenesis (56). Camporesi *et al* (57) demonstrated that HBO therapy may be a viable treatment modality for Ficat stage II ONFH. In the study, symptoms were relieved following a multi-year follow-up, without hip arthroplasty being required. In a study by Reis *et al* (58), 12 patients with Steinberg classification stage 1 ONFH received daily HBO therapy for 100 days. Overall, 81% of the treated patients demonstrated a normal performance on MRI scans compared with 17% in the untreated group. The authors thus presented evidence for the benefit of HBO treatment for idiopathic stage I ONFH. HBO therapy may be useful when carried out in conjunction with core decompression, fenestration and drilling, or other forms of orthopedic intervention. It may also be considered as a primary treatment for ONFH and not merely an adjuvant therapy.

#### Core decompression

The most popular treatment implemented for ONFH is core decompression, which is the standard surgical procedure carried out to treat early-stage non-traumatic ONFH. Various techniques have been trialed in an attempt to discover a treatment to protect the osteonecrotic femoral head. A number of studies have advocated small-diameter percutaneous drilling (59–61). Mont *et al* (62) reported a new technique for core decompression involving multiple small drilled holes and a 3-mm Steinman pin. In the study, 32 of the 45 hips (71%; 35 patients) demonstrated successful clinical results following a mean follow-up of two years (range, 20–39 months). In total, 24 of the 30 stage I hips (80%; 23 patients) had successful outcomes compared with eight of the 15 stage II hips (57%; 12 patients), with no surgical complications. This technique may be effective for delaying the need for total hip arthroplasty in young patients with early (precollapse) stages of ONFH. Certain other studies (62–65) have supplemented core decompression with electrical stimulation.

In a study by Steinberg (66), 116 hips with ONFH underwent decompression and grafting; 74 also received direct current stimulation via a coil inserted into the femoral head. Decompression and grafting were safe and reasonably effective for retarding the progression of ONFH. The supplementary electrical stimulation was observed to further improve the results.

Core decompression remains the leading surgical treatment for ONFH (early- and mid-stage), and the efficacy of any new treatments should be compared with core decompression. Marker *et al* (67) collected data from 1,268 hips following decompression and revealed a clinical success rate of 70% after 63 months, without the need for additional surgery. Certain studies reported core decompression combined with other treatments produces an improved effect for ONFH (68–70). Implanting a demineralized bone matrix or porous hydroxyapatite composite filler achieves more improved results than using core decompression with other augmentation strategies, including bone marrow and growth factors (71–73).

#### Adrenocorticotropic hormone (ACTH) and vascular endothelial growth factor (VEGF)

The development of ONFH is accompanied by the apoptosis of osteocytes and osteoclasts (74). Zaidi *et al* (75) reported that ACTH plays a role in preventing osteonecrosis by promoting osteoblastic support and the expression of VEGF. Early ONFH reveals medium or strong staining of fibroblast growth factor 2 and VEGF, which may promote osteogenesis and bone reconstruction (76).

VEGF is a key factor in bone remodeling. Ingrowth of reparative arterioles has been observed in late-stage osteonecrosis (77). VEGF is a prominent angiogenesis control factor and is used as a therapeutic tool to enhance neovascularization. Angiogenesis is an important part of the bone repair process, and the close connection between blood vessels (endothelial cells) and bone (osteoblasts) was recognized by Trueta and Buhr (78) in 1963. Cao *et al* (79) implanted deproteinized bone (DPB) with VEGF (absorbed in the form of the recombinant plasmid pcDNA3.1-hVEGF165) into necrotic femoral heads to induce angiogenesis and promote repair. The study revealed that transfection with hVEGF165 enhanced local angiogenesis and the DPB-VEGF compound improved repair. Hang *et al* (80) evaluated the efficacy of VEGF165 transgenic bone marrow mesenchymal stem cells on the repair of early-stage ONFH in mature mongrel dogs and demonstrated that the treatment enhanced bone reconstruction and blood vessel regeneration.

### Osteoclast inhibition

#### Pharmacotherapy

Bisphosphonates, such as alendronate, promote osteoclast apoptosis through several mechanisms, including inhibiting protein prenylation and blocking mevalonate metabolism (81). Bisphosphonate reduces edema and the rate of remodeling, which contracts the remodeling spaces and prevents the progression of bone collapse (82). Bone formed during alendronate treatment is histologically normal (83–86) and the treatment appears to offer the greatest protection against fracture. Bisphosphonates are antiresorptive agents that act by inhibiting the action of mature osteoclasts on bone. They transiently stimulate the proliferation and increase the differentiation of pro-osteoblasts, increase the production of the antiresorptive protein osteoprotegerin by osteoblasts, and decrease edema at the site of ONFH, possibly through their anti-inflammatory action.

Agarwala *et al* (87) treated 69 patients with ONFH with alendronate. The disease duration was 0–36 months. The treatment decreased pain and disability within a few weeks and reduced progressive bone collapse, thus positively altering the progress of ONFH. Alendronate is an antiresorptive agent which reduces edema, the risk of fracture, and the rate of remodeling, thereby contracting the remodeling spaces and preventing the progression of bone collapse.

Although bisphosphonates, including alendronate and zoledronate, have revolutionized treatment for ONFH and osteoporosis, a number of studies (88–94) have demonstrated a serious complication of osteonecrosis in the jaw, which has been the subject of much media attention and resulted in patients at risk of ONFH refusing treatment with bisphosphonates. Outeiriño-Fernández (89) reviewed studies on osteonecrosis of the jaw and concluded that alendronate was the bisphosphonate used in the majority of cases, with a mean duration of treatment prior to jaw osteonecrosis of 48.3 months.

In summary, bisphosphonates have revolutionized the treatment of ONFH; however, they carry an increased risk of bisphosphonate-related osteonecrosis of the jaw. Thus, monitoring should be increased in order to prevent the disadvantages associated with these drugs.

## 5. Conclusions

**Figure 1:**
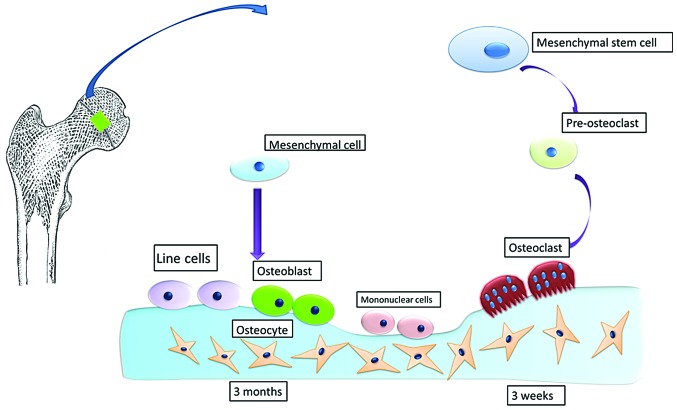
Osteonecrosis of the femoral head. Damage to the bone structure and a reduction in mechanical properties occurs following the beginning of the repair process. It takes ~3 months to build up new bone with effective mechanical properties, but only ~3 weeks for osteoclasts to affect the mechanical strength of the trabecular bone. Thus, the repair process inevitably results in the reduction of the mechanical strength of the femoral head. Collapse of the femoral head occurs under the effect of mechanical load.

ONFH is frequently observed in relatively young adults and its management remains controversial. Conservative treatment may be a major focus for orthopedic studies in the future. The principle of the treatment is to provide mechanical support to prevent collapse of the femoral head and improve the speed and quality of repair at the molecular level, increase osteoclast apoptosis, and reduce osteoblast and osteocyte apoptosis. A number of treatment modalities (operative and nonoperative) have positively altered the natural progression of ONFH during the early stage of the disease. Such treatments include ESWT, HBO, pharmacotherapy and paracentesis therapy with VEGF or stem cells. Surgical approaches include core decompression with structural and cellular supplementation. Damage to the bone structure, a reduction in macromechanical performance, and subsequent collapse is considered to occur during the process of restoration. The phenomenon of the destructive bone repair process is inherent, with bone restoration occurring first followed by repeated action of the bone leading to fatigue from the load and subsequent fracture. Following the initiation of restoration, neovascularization occurs and new bone formation is required to initially break down the sequestrum. Under continued mechanical loads, the femoral head collapses and there is no further bone resorption or osteogenesis. During the repair process, the balanced bone remodeling carried out by osteoblasts and osteoclasts begins. The process of osteogenesis takes ~3 months to establish new bone with an effective mechanical performance; however, it takes only ~3 weeks for osteoclasts to affect the trabecular bone structure. A reduction of the macromechanical strength of the entire femoral head is present in the repair process and also during the collapse of the femoral head under mechanical load. Therefore, delaying or altering the process of femoral head necrosis and collapse through human intervention and recreating a balanced system of osteoblast and osteoclast actions, whilst simultaneously providing sufficient mechanical support, should be studied in the future (Fig. 1).

Preventing collapse of the femoral head is likely to increase in focus in the coming decades since the majority of patients are relatively young at diagnosis. ONFH is undoubtedly a challenging condition to treat; however, ongoing scientific and clinical investigations are progressing towards the development of effective future treatment options.
